# MPNs as Inflammatory Diseases: The Evidence, Consequences, and Perspectives

**DOI:** 10.1155/2015/102476

**Published:** 2015-10-28

**Authors:** Hans Carl Hasselbalch, Mads Emil Bjørn

**Affiliations:** ^1^Department of Hematology, Roskilde Hospital, University of Copenhagen, Køgevej 7-13, 4000 Roskilde, Denmark; ^2^Institute for Inflammation Research, Department of Rheumatology, Rigshospitalet, University of Copenhagen, Blegdamsvej 9, 2100 Copenhagen, Denmark

## Abstract

In recent years the evidence is increasing that chronic inflammation may be an important driving force for clonal evolution and disease progression in the Philadelphia-negative myeloproliferative neoplasms (MPNs), essential thrombocythemia (ET), polycythemia vera (PV), and myelofibrosis (MF). Abnormal expression and activity of a number of proinflammatory cytokines are associated with MPNs, in particular MF, in which immune dysregulation is pronounced as evidenced by dysregulation of several immune and inflammation genes. In addition, chronic inflammation has been suggested to contribute to the development of premature atherosclerosis and may drive the development of other cancers in MPNs, both nonhematologic and hematologic. The MPN population has a substantial inflammation-mediated comorbidity burden. This review describes the evidence for considering the MPNs as inflammatory diseases, *A Human Inflammation Model of Cancer Development*, and the role of cytokines in disease initiation and progression. The consequences of this model are discussed, including the increased risk of second cancers and other inflammation-mediated diseases, emphasizing the urgent need for rethinking our therapeutic approach. Early intervention with interferon-alpha2, which as monotherapy has been shown to be able to induce minimal residual disease, in combination with potent anti-inflammatory agents such as JAK-inhibitors is foreseen as the most promising new treatment modality in the years to come.

## 1. Introduction

Recent studies have provided evidence that the chronic myeloproliferative neoplasms (MPNs), essential thrombocythemia (ET), polycythemia vera (PV), and myelofibrosis (MF), may be preceded by or accompanied by chronic inflammation and also may imply an increased risk for the development of other cancers [1–3]. In these neoplasms morbidity and mortality are massively influenced by cardiovascular and thromboembolic complications [1, 4, 5]. The advanced myelofibrotic stage is typically characterized by transfusion-dependent anemia, large spleen, severe bone marrow fibrosis, and steadily increasing white blood cell counts or severe pancytopenia and end-stage development of acute leukemia, seen in up to 20% of patients with MF [1, 5]. The incidence of MPNs is low, but the prevalence is high and comparable with lung cancer. In 2005, a unique breakthrough was described by the identification of the JAK2V617F mutation in almost all patients with PV and about half of patients with ET and MF [1]. It is possible to monitor the “tumor burden” when analyzing the* JAK2* allelic burden by qPCR. In 2013 the calreticulin mutations were described in a large proportion of the JAK2V617F negative ET and MF patients [6, 7]. The clinical implications of these mutations are being described elsewhere in this Theme Issue.

Chronic inflammation is an important risk factor for the development of atherosclerosis which occurs prematurely in patients with chronic inflammatory diseases, including rheumatoid arthritis, systemic lupus erythematosus, psoriasis, and type II diabetes mellitus. In these diseases, in vivo activation of leukocytes, platelets, and endothelial cells contributes significantly to the increased risk of thrombosis. The same thrombophilia-generating mechanisms are operative in ET, PV, and MF, in which chronic inflammation has recently been described as a potentially very important facilitator not only of premature atherosclerosis, but also of clonal evolution and second cancer [8]. Thus, the chronic MPNs are both “model diseases” for studies of the relationship between chronic inflammation and premature atherosclerosis development in the biological continuum from ET over PV to myelofibrosis and “model diseases” for cancer development from the early cancer stage (ET, PV) to the advanced metastatic cancer stage (MF with myeloid metaplasia) [9–13].

**Figure 1:**
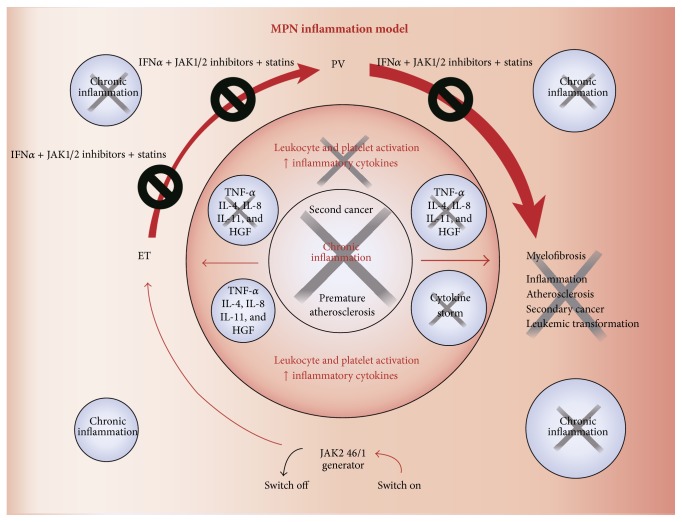
Vicious cycle of inflammation in the biological continuum of ET, PV, and MF. Chronic inflammation is proposed as the trigger and driver of clonal evolution in the biologic continuum from early disease state (ET/PV) to a more advanced disease state (MF). It is possible that combination therapy, using low doses of agents such as interferon-alpha, Janus kinase inhibitors, and statins at the early disease stage, will positively influence the vicious cycle of disease progression. HGF: hepatocyte growth factor; IL: interleukin; MPN: myeloproliferative neoplasm; and TNF: tumor necrosis factor.

Based upon experimental, clinical, and epidemiological studies we herein argue for the MPNs as inflammatory diseases in accordance with the “Human Inflammation Model for Cancer Development.” In the following we will describe the evidence for MPNs as chronic inflammatory diseases and discuss the consequences of chronic inflammation in MPNs in terms of disease progression due to inflammation-mediated clonal expansion and defective tumor immune surveillance. In this context we argue for dampening chronic inflammation at the earliest disease stage (ET/PV), when the tumor burden is minimal, the clone is homogenous (prior to subclone formation and/or acquisition of additional driving mutations), and accordingly the outcome of treatment is logically most favorable (Figure 1).

## 2. The Evidence of a Link between Chronic Inflammation and Cancer

About 30 years ago Dvorak described cancers as “wounds that do not heal,” a concept updated most recently and since 1986 being increasingly recognized [14, 15]. In their seminal contribution from 2000 Hanahan and Weinberg identified the six hallmarks of cancer and recently chronic inflammation was added as the seventh hallmark, emphasizing the huge impact of chronic inflammation on cancer development and progression (“oncoinflammation”) [16, 17]. Accordingly, today chronic inflammation is considered of major importance in the development of cancer and several molecular and cellular signaling circuits have been identified linking inflammation and cancer [18–22]. Indeed, this concept was already described by Virchow in the 19th century when he suggested that chronic inflammation might give rise to malignancy [21]. Regardless, not until more recently, the link between inflammation and cancer has been acknowledged, partly due to epidemiologic studies, which have generated data on chronic infections and inflammation as major risk factors for various types of cancer. In hematological malignancies a link between chronic inflammation and malignant lymphomas has been well described whereas chronic inflammation as a potential initiating event and a driver of clonal evolution in myeloid cancers including MPNs has not been focused upon until very recently [8, 9, 11–13, 23–25].

## 3. The Evidence of MPNs as Inflammatory and Immune Deregulated Diseases

### 3.1. What Is the Epidemiological Evidence?

An increased risk of autoimmune and/or inflammatory conditions has been documented several years ago in patients with myeloid malignancies and recently a large Swedish epidemiologic study concluded that chronic immune stimulation might act as a trigger for the development of the myelodysplastic syndrome (MDS) and acute myelogenous leukemia (AML) [26, 27]. In regard to MPNs, another Swedish study has shown that inflammatory diseases may precede or develop during the course of ET, PV, and MF. In this Swedish study, a prior history of any autoimmune disease was associated with a significantly increased risk of a myeloproliferative neoplasm. The “inflammatory” diseases included, among others, Crohn's disease, polymyalgia rheumatica, and giant cell arteritis, and the “autoimmune” diseases included immune thrombocytopenic purpura and aplastic anemia [2]. The 46/1 haplotype is present in 45% of the general population and is associated with a predisposition to acquire the* JAK2*V617F mutation and accordingly MPNs but also predisposes to MPNs with no mutation of* JAK2* and to MPNs with mutation in* MPL* [28–31]. Importantly, epidemiological studies have shown that the frequency of the* JAK2* 46/1 haplotype is increased in inflammatory diseases, including Crohn's disease [32, 33].

Risk factors for developing atherosclerosis, a chronic inflammatory disease, have been investigated in a large Danish epidemiological study of 49 488 individuals from the Copenhagen General Population Study. It was discovered that those harboring the* JAK2*V617F mutation had a 2.2-/1.2-fold risk (prevalent/incident) of ischemic heart disease [34].

### 3.2. What Is the Histomorphological Evidence?

Already about 40 years ago it was speculated if autoimmune bone marrow damage might be incriminated in the pathogenesis of “idiopathic myelofibrosis” (IMF). Several observations seem to support the participation of immune mechanisms in the development of bone marrow fibrosis. Thus, histopathological findings of “Fibrin-Faser-Stern” figures, increased numbers of plasma cells and lymphocytes with plasmacytoid appearance, the demonstration of a parallel increase in interstitial deposits of immunoglobulins and the extent of bone marrow fibrosis, and the development of bone marrow fibrosis after repeated antigen injections in animal models all render immune-mediated bone marrow fibrosis possible [35–41]. Importantly, the findings of “Fibrin-Faser-Stern” figures and lymphoid aggregates in bone marrows from MPNs patients have been variably interpreted as evidence of immune activity in the marrow with deposition of immune complexes [35–38]. Immune activity in the bone marrow with an increase of lymphoid nodules has been found to be most prominent in the early stage of IMF [37, 38]. A most recent study investigated the mechanism of bone marrow fibrosis in patients with MF by comparing TGF-*β*1 signaling of marrow and spleen cells from patients with MF and of nondiseased individuals. The expression of several TGF-*β*1 signaling genes was altered in the marrow and spleen of MF patients, respectively. Abnormalities included genes of TGF-*β*1 signaling, cell cycling, and Hedgehog and p53 signaling. Pathway analysis of these alterations predicted an increased osteoblast differentiation, ineffective hematopoiesis, and fibrosis driven by noncanonical TGF-*β*1 signaling in the marrow and increased proliferation and defective DNA repair in the spleen. The hypothesis that fibrosis in MF might result from an autoimmune process, triggered by dead megakaryocytes, was supported by the findings of increased plasma levels of mitochondrial DNA and anti-mitochondrial antibodies in MF patients. It was concluded that autoimmunity might be a plausible cause of marrow fibrosis in MF [42]. Finally, the clinical observations of a favorable outcome of immunosuppressive therapy in some MF patients with evidence of autoimmune activity support the concept that autoimmunity, immune dysfunction, and chronic inflammation may be important factors in pathogenesis [43–48].

### 3.3. What Is the Clinical Evidence?

#### 3.3.1. The Inflammation-Mediated Cardiovascular and Thromboembolic Disease Burden

**Figure 2:**
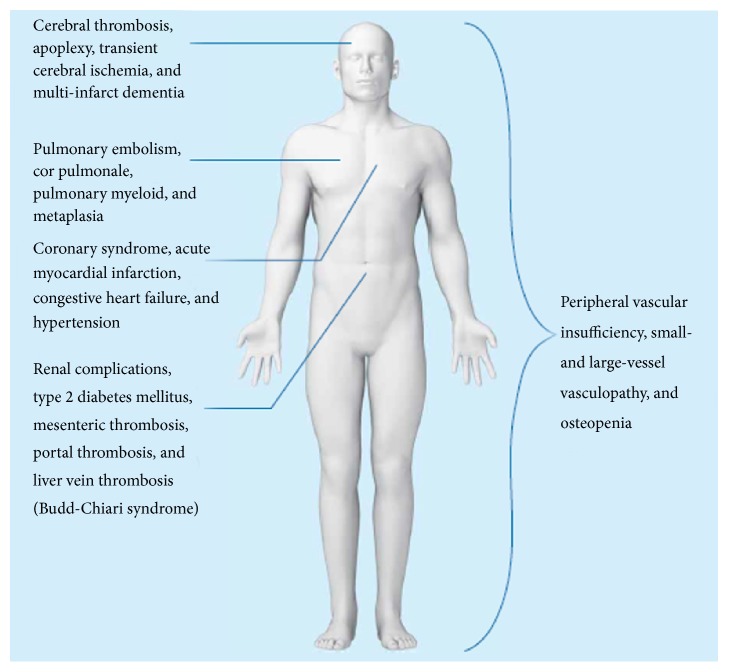
Patients with MPNs have a massive cardiovascular and thromboembolic disease burden.

Patients with MPNs have a massive cardiovascular disease burden with a high risk of thrombosis (Figure 2), which is partly explained by excessive aggregation of circulating leukocytes and platelets due to in vivo leukocyte-platelet and endothelial activation in combination with a thrombogenic endothelium [1, 4]. In addition MPNs are associated with a procoagulant state, which has recently been elegantly reviewed by Barbui et al. [49]. The hyperactivation of circulating cells in MPNs has been thought to be attributed to the clonal myeloproliferation. Thus, the* JAK2*V671F mutation per se has been shown to induce leukocyte and platelet activation and several clinical studies have demonstrated that* JAK2*V617F positivity is a thrombogenic factor in MPNs [49–52]. Of note, Barbui et al. have recently shown that the level of C-reactive protein (CRP) is elevated in patients with ET and PV and correlates significantly with the* JAK2*V617F allele burden [53]. Furthermore, elevated CRP levels have also been associated with shortened leukemia-free survival in myelofibrosis [54]. It was speculated if sustained inflammation might elicit the stem cell insult by inducing a state of chronic oxidative stress with elevated levels of reactive oxygen species (ROS) in the bone marrow, thereby creating a high-risk microenvironment for induction of mutations in hematopoietic cells [9]. Being a sensitive marker of inflammation and influencing, for example, endothelial function, coagulation, fibrinolysis, and plaque stability, CRP is considered to be a mediator of vascular disease and accordingly a major vascular risk factor as well [55–57]. This association has recently been demonstrated in a meta-analysis, showing continuous associations between the CRP concentration and the risk of coronary heart disease, ischemic stroke, and vascular mortality [58]. For decades it has been known that atherosclerosis and atherothrombosis are chronic inflammatory diseases [59, 60]. Several studies have reported that chronic inflammatory diseases (e.g., rheumatoid arthritis, psoriasis, systemic lupus erythematosus, and diabetes mellitus) are associated with accelerated atherosclerosis and accordingly development of premature atherosclerosis (early ageing?) [61–65]. In addition, considering the association between atherosclerosis and venous thrombosis, chronic inflammation indirectly predisposes to venous thrombosis and pulmonary thromboembolism as well [66]. In the context of the associations between inflammation and CRP in ET and PV, inflammation might be considered to be a secondary event elicited by clonal cells [53]. However, elevated leukocyte and platelet counts in MPNs may not only reflect clonal myeloproliferation but also reflect the impact of chronic inflammation per se on the clonal cells. In particular, this interpretation is intriguing when considering that one of the hallmarks of MPNs is inherent hypersensitivity to growth factor and cytokine stimulation [8]. In this perspective, chronic inflammation in MPNs may also have a key role in promoting premature atherosclerosis and all its debilitating cardiovascular and thromboembolic complications, the common denominators for their development being elevated leukocyte and platelet counts, elevated CRP levels, and in vivo leukocyte-platelet and endothelial cell activation, taking into account that platelet-leukocyte interactions link inflammatory and thromboembolic events in several other inflammation-mediated diseases [67].

#### 3.3.2. Inflammation-Mediated Chronic Kidney Disease

Uncontrolled chronic inflammation is associated with organ dysfunction, organ fibrosis, and ultimately organ failure [68]. This development is classically depicted in patients with the metabolic syndrome progressing to type II diabetes mellitus (DM) which, without adequate treatment to normalize elevated blood glucose levels, may rapidly develop organ failure due to accelerated atherosclerosis (e.g., hypertension, ischemic heart disease, stroke, dementia, peripheral arterial insufficiency, venous thromboembolism, and chronic kidney disease). The progressive deterioration of multiple organs in uncontrolled DM consequent to elevated blood glucose levels with in vivo leukocyte-platelet and endothelial activation and development of premature atherosclerosis is in several aspects comparable to the multitude of systemic manifestations in patients with uncontrolled MPNs, the common denominators being a huge cardiovascular disease burden and thromboembolic complications [10]. Importantly, similar to patients with type II DM, it has been demonstrated that patients with MPNs have an increased risk of developing chronic kidney disease [69]. It was concluded that progressive renal impairment may be an important factor in MPNs contributing to the comorbidity burden and likely to the overall survival. In addition it was speculated whether chronic inflammation with accumulation of ROS might be a driving force for impairment of renal function and accordingly supportive of early intervention in order to normalize elevated cell counts and reduce the chronic inflammatory drive elicited by the malignant clone itself [69].

#### 3.3.3. Inflammation-Mediated “Autoinflammatory” Diseases

As outlined above, patients with MPNs may have an increased risk of various autoimmune, “autoinflammatory,” or inflammatory diseases. Thus, associations have been reported with systemic lupus erythematosus, progressive systemic sclerosis, primary biliary cirrhosis, ulcerative colitis, Crohn's disease, nephrotic syndrome, polyarteritis nodosa, Sjögren syndrome, juvenile rheumatoid arthritis, polymyalgia rheumatica/arteritis temporalis, immune thrombocytopenic purpura (ITP), and aplastic anemia. In large epidemiological studies these associations have only been significant for Crohn's disease, polymyalgia rheumatica/arteritis temporalis, and ITP [2]. Interestingly, a particular subtype of myelofibrosis, “primary autoimmune myelofibrosis,” has been described. This subtype has been considered to be a nonclonal and nonneoplastic disease, featured by anemia/cytopenias and autoantibodies suggesting systemic autoimmunity. Most patients have no or only mild splenomegaly and the bone marrow biopsy exhibits MPN-like histology with fibrosis, hypercellularity, and megakaryocyte clusters. In addition, bone marrow lymphoid aggregates are prominent [46]. It remains to be established if this subset of MF actually exists or if these patients indeed should be categorized within the MPNs disease entity, taking into account that autoimmunity and chronic inflammation today are considered to have a major role in MPNs pathogenesis.

#### 3.3.4. Inflammation-Mediated Osteopenia

A recent Danish registry study has shown that patients with ET and PV have an increased incidence of fractures compared with the general population [70]. Taking into account that chronic inflammation has been suggested to explain the initiation of clonal development and progression in chronic myeloproliferative neoplasms and other chronic inflammatory diseases that are associated with an increased risk of osteopenia it has been speculated if chronic inflammation might induce osteopenia in MPNs and by this mechanism also predispose to the increased risk of fractures [12, 70–72].

#### 3.3.5. Inflammation-Mediated Second Cancers

As noted previously patients with MPNs have been shown to have an increased risk of second cancers [3, 5]. In the perspective that chronic inflammation may be a driving force for clonal evolution in MPNs it is intriguing to consider if chronic inflammation may contribute to the development of second cancers in MPNs as well, taking into account the close association between inflammation and cancer [8, 9, 11–13, 17–22]. In this regard a defective “tumor immune surveillance” consequent to immune deregulation, which has been demonstrated in MPNs in several recent studies and most recently comprehensively reviewed, might be of importance [42, 73–75]. Of note, the increased risk of second cancers has also been recorded prior to the MPNs diagnosis emphasizing that the MPNs may have a long prediagnosis phase (5–10–15 years) with a chronic inflammatory state promoting mutagenesis, defective tumor immune surveillance, and immune deregulation [9, 76–78]. This concept is compatible with the most recent observations of additional mutations that are already present at the time of diagnosis likely induced by a sustained inflammatory drive on the malignant clone several years before diagnosis [7, 9, 78, 79] (Figure 4).

### 3.4. What Is the Biochemical Evidence?

As outlined above MPNs are associated with a low-grade inflammatory state as assessed by slightly elevated CRP in a large proportion of patients with ET and PV [53]. The CRP levels are steadily increasing when patients enter the accelerated phase towards leukemic transformation [54]. Considering the close association between CRP and other inflammatory markers, the leukocyte and platelet counts, it is most relevant to speculate if leukocytosis and thrombocytosis in MPNs are also attributed to the chronic inflammatory drive per se with sustained generation of inflammatory products that fuel the malignant clone in a vicious self-perpetuating circle [8, 11]. Similar to CRP, plasma fibrinogen and plasma D-dimers levels are slightly elevated in several patients and may indeed be more sensitive inflammatory markers than CRP (unpublished observations). Proinflammatory cytokines are elevated in a substantial proportion of patients with MPNs, a topic which has recently been reviewed and thoroughly described by Fleischman and others in this Theme Issue [11].

The hypothesis and the concept of MPNs and the advanced MF stage being elicited and perpetuated by autoimmune/inflammatory mechanisms were intensely investigated and discussed already 30 years ago. Some of the clinical and histomorphological issues with associations between MPNs and autoimmune/inflammatory states have already been addressed above. In addition, several studies from that period reported biochemical evidence of autoimmunity/inflammation in MPNs, such as elevated levels of antibodies to RBCs, antibodies to platelets, anti-nuclear and anti-mitochondrial antibodies (ANA and AMA), rheumatoid factor, lupus-like anticoagulant, low levels of complement, complement activation, increased levels of immune complexes (ICs), and increased levels of interleukin-2 soluble receptors (s-IL2R) [38, 43, 80–85]. It was debated whether deposition of immune complexes in the bone marrow, either formed in situ or trapped from the circulation, might be followed by complement activation with subsequent local inflammatory reaction, an interpretation fitting very well with the findings of complement activation in MF patients [80, 81]. Of note, circulating immune complexes were predominantly found in the early disease stage. Since circulating ICs were in some studies mainly found in MF patients with a short duration of disease from diagnosis it was hypothesized that potential immune-mediated bone marrow damage might indeed occur in the early phase of the disease and the late, fibrotic stage with undetectable IC representing the “burnt out” phase of the disease [81, 84]. Today, 30 years after the detection of IC in MPNs, their significance in MF and related neoplasms remains unsettled. With the renaissance of the concept of autoimmune bone marrow damage and chronic inflammation as driving forces for disease evolution and progression further studies on circulating ICs and their pathogenetic and clinical relevance are highly relevant and timely. Indeed, their detection may reflect ongoing inflammatory immune reactions in the circulation and in the bone marrow, being likely most pronounced in the initial disease phase and possibly related to a more acute course of the disease [81]. Most recently, a comprehensive study of autoimmune phenomena and cytokines in 100 patients with MF, including early stage MF, has added further proof of the concept that autoimmune and inflammatory mechanisms may be highly important in the pathogenesis of MPNs [86]. Importantly, organ/non-organ-specific autoantibodies were found in 57% of cases, without clinically overt disease, and mostly in low-risk/intermediate-risk-1 and MF-0/MF-1. Furthermore, TGF-*β* and IL-8 were increased in MS-DAT positive cases, and TGF-*β* and IL-17 were elevated in early clinical and morphological stages, while IL-8 increased in advanced stages. It was concluded that autoimmune phenomena and cytokine dysregulation may be particularly relevant in early MF [86].

Several studies have shown that circulating YKL-40 levels are elevated in a number of different diseases, including cancer, diabetes mellitus, and cardiovascular diseases, in which YKL-40 serves as an excellent marker of the disease burden. Importantly, a state of chronic inflammation is shared by them all, and YKL-40 also has a major impact upon the severity of chronic endothelial inflammation, which today is considered of crucial importance for the development of atherosclerosis. Considering the MPNs as chronic inflammatory diseases and accordingly with an increased risk of development of premature atherosclerosis we hypothesized that circulating YKL-40 might be an ideal marker of the integrated impact of chronic inflammation in MPNs and accordingly might display correlations with conventional markers of inflammation and disease burden in MPNs. Indeed, we have recently shown that circulating YKL-40 is a potential novel biomarker of disease activity and the inflammatory state in myelofibrosis and related neoplasms [87, 88]. These studies have demonstrated a steady increase in YKL-40 from early cancer stage (ET) over PV to the advanced cancer stage with myelofibrosis, which exhibited the highest YKL-40 levels of them all. Highly interesting, we also found a significant correlation between YKL-40 and several markers of inflammation and disease burden, including neutrophils, platelets, CRP, LDH, and the* JAK2*V617F allele burden. Accordingly, circulating YKL-40 may be a novel marker of inflammation, disease burden, and progression in MPNs [87, 88].

### 3.5. What Is the Molecular Evidence?

**Figure 3:**
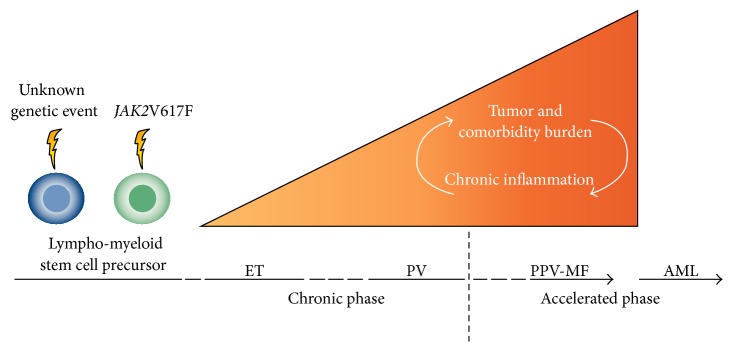
Chronic inflammation as the driving force for clonal evolution in MPNs. Tumor burden and comorbidity burden are illustrated for patients with* JAK2*V617F positive MPNs. Comorbidity burden increases from early disease stage (ET/PV) to the accelerated phase with myelofibrotic and leukemic transformation. With permission: H. C. Hasselbalch [12]. AML: acute myeloid leukemia; ET: essential thrombocythemia; JAK: Janus kinase; PPV-MF: post-polycythemia vera; and PV: polycythemia vera.

**Figure 4:**
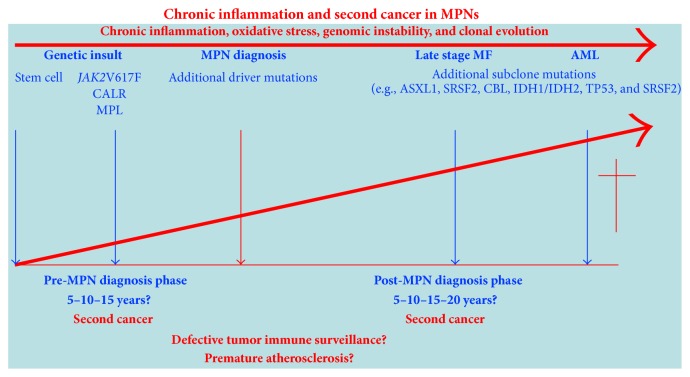
Patients with MPNs have an increased risk of second cancer not only after the MPNs diagnosis but also in the pre-MPNs diagnosis phase, which may last several years in which the patients are at an increased risk of severe cardiovascular and thromboembolic events. According to this model, the initial stem cell insult has occurred 5–10–15 years before the MPNs diagnosis.

The concept of chronic inflammation leading to clonal evolution in MPNs is also supported by gene expression profiling studies (Figure 3), which have unraveled deregulation of several genes that might be implicated in the development and phenotype of the MPNs [89–92]. Using whole-blood transcriptional profiling and accordingly obtaining an integrated signature of genes expressed in several immune cells (granulocytes, monocytes, B cells, T cells, and platelets), we have shown that the MPNs exhibit a massive upregulation of IFN-related genes, particularly interferon-inducible (IFI) gene IFI27 and severe deregulation of other inflammation and immune genes as well. Indeed, several genes (e.g., IFI27) displayed a stepwise upregulation in patients with ET, PV, and PMF with fold changes from 8 to 16 to 30, respectively. The striking deregulation of IFI genes may likely reflect a hyperstimulated but incompetent immune system being most enhanced in patients with advanced MF. In this context, the massive upregulation of the IFI27 gene may also reflect an exaggerated antitumor response as part of a highly activated IFN system, including enhanced IFN gamma expression, which might also imply activation of dendritic cells. IFI27 is also upregulated during wound repair processes, which may be of particular relevance when considering the Dvorak thesis on “Tumors: wounds that do not heal” [14, 15]. Thus, it is tempting to argue that MPNs are “wounds in the bone marrow that will not heal,” owing to the continuous release from clonal cells of growth factors and matrix proteases with ensuing extracellular remodeling of the bone marrow stroma. In this scenario, one might speculate whether the high expression of IFI27 may reflect these processes as well, IFI27 cooperating with distinct genes of potential importance for egress of CD34^+^ cells from the bone marrow niches into the circulation [93]. In the context of matrix remodeling during cancer metastasis (which in MPNs consists of egress of CD34^+^ cells from the bone marrow niches into the circulation) it is of particular interest to note that IFN-inducible genes, including IFI27, have been shown to be associated with the so-called metagenes in patients with breast cancer, accurately identifying those patients with lymph node metastasis and accordingly predictors of outcomes in individual patients [94]. Thus, the highly upregulated IFI27 gene in MPNs may reflect progressive clonal evolution with “metastasis” (extramedullary hematopoiesis) despite an exaggerated yet incompetent IFN-mediated antitumor response by activated dendritic cells and T cells. In this regard a hyperstimulated immune system might also contribute to the increased risk of autoimmune diseases in MPNs. Accordingly the interferon signature may reflect MF as the terminal stage of chronic inflammation with a huge burden of oxidative stress, genomic instability, and accumulation of additional inflammation-induced mutations, the ultimate outcome being leukemic transformation [8–12]. During this evolution from early cancer stage to the metastatic stage with MF, the interferons are important cytokines for immunity and cancer immunoediting [95]. For this and several other reasons IFN is, today and in the future, considered the cornerstone in the treatment of MPNs which, when instituted in the very early disease stage, may be able to quell the fire and accordingly induce “minimal residual disease” and in some patients likely cure as will be discussed below [96–103]. Supporting chronic inflammation as the driving force for clonal evolution is also the most recent whole-blood gene expression studies, showing a marked deregulation of oxidative stress genes in MPNs [104]. This issue is extensively described by Bjørn and Hasselbalch in the chapter on “The Role of Reactive Oxygen Species in Myelofibrosis and Related Neoplasms.”

### 3.6. What Are the Consequences of Chronic Inflammation in MPNs?

#### 3.6.1. The Bone Marrow Is Burning

In MPNs chronic inflammation may elicit a “cytokine storm,” “a wound that does not heal,” due to the continuous release of proinflammatory cytokines that in a self-perpetuating vicious circle drives the malignant clone. Importantly, in this inflammatory micromilieu, reactive oxygen species (ROS) are steadily accumulating, giving rise to increasing genomic instability, subclone formation with additional mutations, and ultimately bone marrow failure as a consequence of inflammation-mediated ineffective myelopoiesis (anemia, granulocytopenia, and thrombocytopenia), accumulation of connective tissue in the bone marrow, and ultimately leukemic transformation [8, 9, 11–13]. The impact and consequences of ROS for disease progression have been thoroughly described elsewhere by Bjørn and Hasselbalch and the impact of chronic inflammation on bone marrow stroma has been reviewed by Marie Caroline Le Bousse Kerdiles and coworkers.

Chronic inflammation in the bone marrow microenvironment may enhance in vivo granulocyte activation with ensuing release of a vast amount of proteolytic enzymes from neutrophil granules, thereby facilitating egress of CD34^+^ cells and progenitors from bone marrow niches into the circulation (“metastasis”).

#### 3.6.2. The Spleen Is Burning

A common complaint in MPNs patients with enlarged spleens is a “burning” spleen, which on clinical examination may also be extremely painful. Although spleen infarction may occasionally explain the spleen pain, it is in the large majority of patients attributed to inflammation as evidenced by a remarkable relief when being treated with high-dose glucocorticoids and, in particular, during treatment with JAK2 inhibitors which within a few days is associated with a reduction in spleen size and a concomitant improvement in spleen pain as well. Accordingly, the rapid reduction in spleen size during, for example, treatment with ruxolitinib, is primarily consequent to its very potent anti-inflammatory effects as also evidenced by the rapid decrease in circulating inflammatory cytokines [11, 12].

#### 3.6.3. The Circulation Is Burning

**Figure 5:**
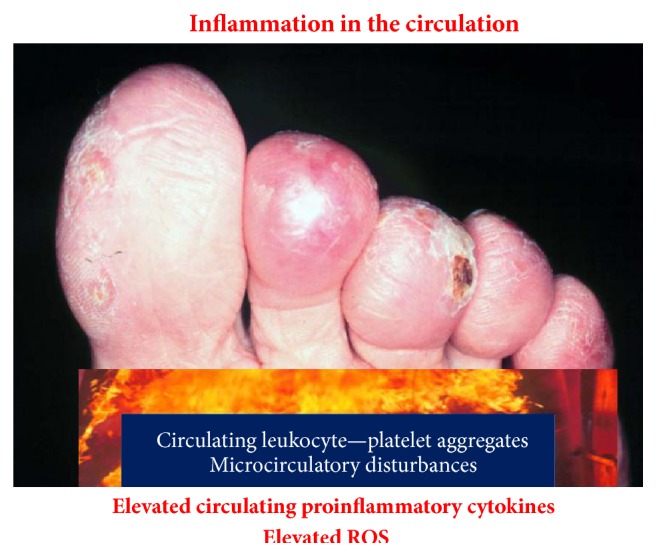
Inflammation in the circulation elicits in vivo leukocyte and platelet aggregation giving rise to circulating microaggregates with ensuing impairment of microcirculation, tissue ischemia, and ultimately development of ulcers on toes and fingers which may terminate with gangrene. Treatment with aspirin momentarily resolves microaggregation with improvement in microcirculation.

As outlined above circulating levels of a large number of inflammatory cytokines are elevated in patients with MPNs [11, 105, 106]. These cytokines activate circulating leukocytes and platelets and also activate endothelial cells as well, giving rise to aggregation of leukocytes and platelets with the formation of microaggregates that compromise the microcirculation in several organs [48, 51] (Figure 5). Taking into account that a large proportion of the circulating leukocytes and platelets are activated per se due to their clonal origin the additional impact of chronic inflammation upon in vivo activation of these cells may profoundly worsen the microcirculation in several organs with ensuing tissue ischemia and associated symptoms, including, for example, CNS-related symptoms (headaches, visual disturbances, dizziness, infarction, and dementia), pulmonary symptoms (dyspnoea due to pulmonary embolism, inflammation due to sequestration of leukocytes and platelets and megakaryocytes in the microcirculation with release of a large number of inflammatory products), symptoms of ischemic heart disease (angina, infarction, and congestive heart failure), or symptoms of peripheral vascular insufficiency [4, 5, 12, 34, 107–112] (Figure 5).

## 4. Discussion and Perspectives

The perspectives of the MPNs as “A Human Inflammation Model for Cancer Development” being driven by chronic inflammation in a self-perpetuating vicious circle from early cancer stage (ET/PV) to the advanced “metastatic” stage with severe MF and egress of CD34^+^ cells from bone marrow niches to the circulation (metastasis to the spleen and liver and elsewhere) are several [8–13, 96–103].

**Figure 6:**
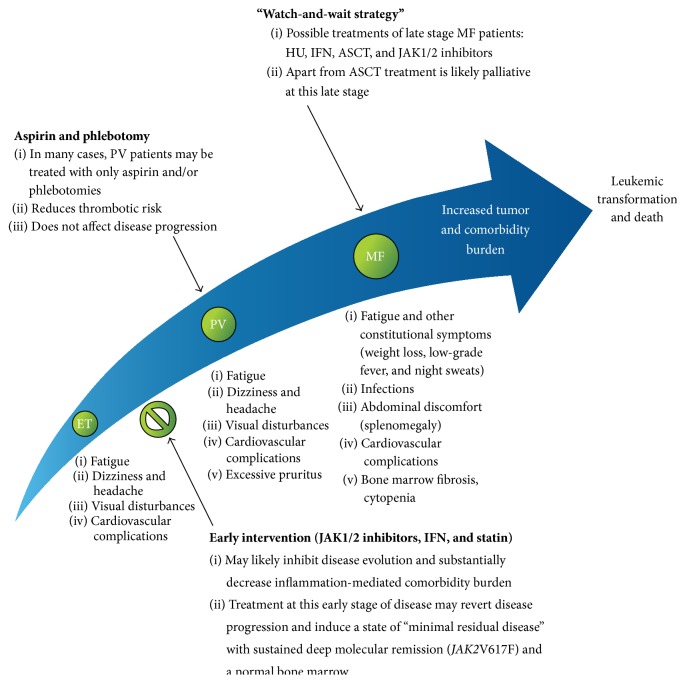
The MPNs care pathway and the effect of early intervention. It is suggested that ET, PV, and MF form a biological continuum and, thus, early intervention with combination therapies including JAK1/2 inhibitors, IFN, and/or statins is likely to result in the inhibition of disease evolution. ASCT: allogeneic stem cell transplantation; ET: essential thrombocythemia; HU: hydroxyurea; IFN: interferon; JAK: Janus kinase; MF: myelofibrosis; and PV: polycythemia vera (with permission: H. C. Hasselbalch [12]).

Firstly, this novel concept calls for the urgent need of a fundamental change in our therapeutic attitude from the conventional “watch-and-wait strategy” to “the early intervention concept” using interferon-alpha2 (IFN) as the cornerstone in the early treatment from the time of diagnosis [96–103] (Figures 1 and 6). However, since access to IFN for the routine use in patients with MPNs is highly variable, a prerequisite for such a change is that opinion leaders within the international MPNs scientific community realize that the time has come to rethink when, how, and who we should treat with IFN. Today the world is divided into two: in one world, not having access to IFN and accordingly its MPNs experts no or only modest experience with the use of IFN most ET and PV patients are followed according to the “watch-and-wait strategy,” receiving only cytoreductive treatment with hydroxyurea (HU) for elevated cell counts if they have suffered a prior thrombosis, the platelet count being >1500 × 10^9^/L or if they are elderly (>60 years) [113–120]. This risk stratification therapy is partly based upon the concept “do no harm to the patient,” since HU treatment implies an increased risk of skin cancer and an increasing concern in regard to an increased risk of other cancers as well, including myelodysplasia and acute myelogenous leukemia [98, 100, 102, 121–125]. Accordingly, in this part of the world, HU is avoided in younger patients with ET and PV, who then may not receive cytoreductive treatment for elevated leukocyte counts or elevated platelet counts (>1500 × 10^9^/L) unless they experience the catastrophe, thrombosis or major hemorrhage and consequent* sequelae*. In the other world, having access to IFN, most newly diagnosed patients with ET, PV, and hyperproliferative myelofibrosis are treated routinely with low-dose IFN as described in several studies and reviews during recent years [96–103].

Secondly, we, the MPNs scientific community, and health authorities (Food and Drug Administration (FDA) and EMA (European Medical Agency)) also need to rethink if optimal treatment of MPNs is only determined by the randomized trial or if optimal treatment might also be determined by several single-arm studies proving safety and efficacy of oncology drugs in orphan diseases [102, 123]. In this regard IFN in MPNs is a classic example which for sure has shown safety and efficacy in a large number of clinical studies during the last 25 years but, regardless, is still being considered experimental or not evidence-based therapy, in the world without access to IFN.

Accordingly, promotion of rapidly accumulating evidence for the concept of MPNs as “A Human Inflammation Model for Cancer Development” into clinical practice with upfront treatment with IFN to inhibit clonal expansion (“stopping the fuel that feeds the fire”) requires a global signature from the MPN scientific community, a fusion of the two worlds, and an urgent action from health authorities to accept that approval of a drug for orphan diseases—IFN in MPNs—is applicable when safety and efficacy have been demonstrated in a large number of single-arm studies during the last 2 decades [102, 126].

Thirdly, the proof of concept that chronic inflammation may elicit MPNs needs to be further investigated in other mouse models than the ones already published, including the MPN-mouse model from Heike Pahl's group and the mouse model that has displayed formaldehyde (FA) by inhalation to be able to induce inflammation and ROS accumulation in the bone marrow with ensuing MPN-like blood and bone marrow features such as anemia, leukopenia and thrombocythemia, and megakaryocyte hyperplasia with myelofibrosis, respectively [13, 127, 128].

Fourthly, considering chronic inflammation as a potential trigger of MPNs evolution and the experimental proof that FA induces inflammation in the bone marrow with myelofibrosis, it is indeed intriguing to speculate if cigarette smoke that contains thousands of toxic inflammatory agents, including FA, may actually be a risk factor for development of MPNs [129]. Thus, smoking is associated with elevated hematocrit, leukocytosis, monocytosis, and occasionally thrombocytosis—all are hallmarks in patients with MPNs. To this end the JAK-STAT and NF-kB signalling pathways are activated in both smokers and in patients with MPNs. Additionally, both share elevated levels of several proinflammatory cytokines, in vivo activation of leukocytes and platelets, endothelial dysfunction, and increased systemic oxidative stress. Indeed, smoke as a chronic inflammation stimulus giving rise to a chronic myelomonocytic response and ultimately MPNs fits very well with the excellent inflammation model for MPNs development as recently described by Hermouet and coworkers [31]. Accordingly, there is reason to believe that smoking may be both a trigger for and a driver of clonal evolution in MPNs taking into account that both smoking and MPNs are associated with chronic inflammation and systemic oxidative stress. In this context smoking may augment chronic inflammation in MPNs, thereby magnifying the risk of thrombosis, clonal expansion, and second cancers. The role of smoking in MPNs pathogenesis is further supported by a most recent study showing that a high proportion of MPNs patients actually have a smoking history [130]. An association between smoking and MPNs evolution is also supported by the fact that the most frequent second cancers in patients with MPNs are lung and urinary tract cancers which are most prevalent in smokers [3].

Fifthly, chronic systemic inflammation in patients with MPNs may predispose to or aggravate existing inflammation-mediated diseases in MPNs patients. Thus, it might be anticipated that chronic inflammation associated with (other) chronic inflammatory diseases, for instance, inflammatory rheumatological or dermatological diseases (e.g., polymyalgia rheumatica, rheumatoid arthritis, psoriasis, hidradenitis, and systemic lupus erythematosus), chronic inflammatory bowel diseases (Crohn's disease, colitis ulcerosa), chronic obstructive pulmonary disease and cancers (e.g., lung cancer) might ultimately elicit MPNs in a subset of the patients consequent to the chronic inflammation-mediated myelomonocytic drive [31]. Importantly, in these patients, anemia, leukocytosis, and thrombocythemia are ascribed to their chronic inflammatory disease or cancer and, accordingly, they are not normally screened for JAK2V617F, CALR, or MPL mutations. In the context of MPNs as inflammatory diseases, being potentially triggered and driven by chronic inflammation, the time is ripe to consider if the above disease categories should be investigated more rigorously for MPNs than being clinical practice today. Indeed, such studies are urgently needed to elucidate and expand the role of chronic inflammation as a true trigger for and driver of clonal evolution in MPNs.

Sixthly, chronic inflammation and oxidative stress may have therapeutic implications. Thus, it might be anticipated that patients with systemic chronic inflammation due to concurrent inflammation-mediated comorbidities may exhibit an inferior response to cytoreductive therapy necessitating higher dosages of, for example, hydroxyurea to obtain normal leukocyte and platelet counts. Furthermore, the response to IFN might be impaired considering that IFN signalling is impaired by inflammation and oxidative stress [131].

Seventhly, in the context that “triple-negative” (negative for JAK2V617F, CALR, and MPL-mutations) ET patients have a much more favourable prognosis than mutation-positive ET patients, some triple-negative “ET” patients may actually not have a MPNs but instead polyclonal inflammation-driven thrombocythemia. If so, the subset of “triple-negative” “ET” patients may be associated with a heavy comorbidity burden of chronic inflammatory diseases, an issue which deserves to be investigated systematically.

Eighthly, by dampening chronic inflammation using potent anti-inflammatory agents such as JAK2 inhibitor treatment and statins, it is anticipated that the rate of thromboembolic events will likely decline, since chronic inflammation per se carries an increased risk of thrombosis due to several factors as outlined above (leukocytosis, thrombocytosis, and in vivo leukocyte-platelet and endothelial activation). This issue on inflammation-mediated thrombogenesis has been dealt with most recently [132].

Ninthly, chronic inflammation in MPNs, if left untreated with elevated platelet counts, may worsen the prognosis of second cancers, which MPNs patients are prone to develop, not only after the MPNs diagnosis but also prior to the diagnosis [3, 76]. This particular issue, the “Platelet-Cancer-Loop” in MPNs, and the perspectives for prognosis of second cancers when not treating elevated platelet counts in MPNs have most recently been reviewed and debated [78, 133]. Indeed, elevated platelet counts in MPNs may contribute to the inferior prognosis of second cancers in these patients, most recently being reported in a large Danish epidemiological study [134].

Tenthly, the notion of treating these diseases only when far advanced is antithetical to treating other forms of cancer. The model of clonal evolution, the occurrence of additional molecular abnormalities, and the development of metastatic sites of disease following extramedullary hematopoiesis of CD34^+^ cells in the spleen and liver are just some of the compelling reasons to consider treating sooner rather than later, when the tumor burden is less rather than more and before disease progression occurs. The fact that both rIFN and JAK1/2 inhibition can cause molecular change in JAK2V617F allele burden and revert cytogenetic and other clonal abnormalities adds impetus to this argument. From the perspective that chronic inflammation may drive clonal expansion in these neoplasms early treatment may induce a state of minimal disease in a substantial number of patients. This may alter the natural history of the MPNs and the otherwise inevitable path towards thrombosis, irreversible MF, and leukemic transformation [97–103].

Eleventh, statins have, in addition to a cholesterol-lowering effect, many so-called pleiotropic effects, including antiproliferative, proapoptotic, antiangiogenic, antithrombotic, and especially potent anti-inflammatory effects [135]. Most recently, it has been shown that statins also significantly inhibit the malignant MPNs cell growth, including a potent synergistic effect with JAK inhibition [136, 137]. Thus, the perspectives may be that statins will achieve an important role in the future MPNs treatment in combination with JAK1/2 inhibitors and IFN-alpha2, a combination therapy, which—if instituted already from the time of diagnosis by potent inhibition of clonal proliferation and hence blockage of chronic inflammation generated by the malignant clone itself—may envisage the hope of reverting MPNs disease progression by inhibiting inflammation-driven genomic instability, subclone formation, mutagenesis, and thereby the ultimate transformation to myelofibrosis and acute myeloid leukemia. In regard to the anti-inflammatory, antithrombotic, and cytoreductive potential of statins and most lately the epidemiological evidence that statins reduce cancer-related mortality the rationale for the use of statins in patients with MPNs—per se accompanied by an increased risk of second cancers with an inferior prognosis—is only further supported [134–138]. Taking into account that MPNs patients may be prone to develop inflammation-mediated osteopenia with an increased risk of fractures early diagnosis and treatment of osteopenia with bisphosphonates may be an option in the future. Indeed, bisphosphonates also possess potent anti-inflammatory, immunomodulatory anticancer properties and may have a synergistic effect with statins in targeting the bone marrow stroma niche, thereby inhibiting the egress of CD34^+^ cells from stem cell niches [139]. To this end, several reports have documented beneficial effects of treatment with bisphosphonates in MPNs [140–145]. The rationales for the mevalonate pathway as a therapeutic target in the treatment of MPNs have been thoroughly described in recent reviews [135, 146].

## 5. Conclusion

The concept of chronic inflammation as a major driver of disease progression in MPNs opens the avenue for clinical trials in which the two most promising agents within MPNs—IFN and ruxolitinib—are combined and instituted in the early disease stage according to the early intervention concept. The proof of concept and the rationales for this combination therapy have most recently been published [147] and a Danish study on combination therapy with low-dose pegylated IFN and ruxolitinib is ongoing with very promising preliminary results. The ability of IFN to induce deep molecular responses with normalisation of the bone marrow, even years after cessation of IFN, and the role of inflammation in the initiation and progression of MPNs make the combination of IFN and ruxolitinib one of the most promising new treatment strategies for patients with MPNs [8, 9, 11–13].
